# Integrating AI Segmentation, Simulated Digital Twins, and Extended Reality into Medical Education: A Narrative Technical Review and Proof-of-Concept Case Study

**DOI:** 10.3390/jpm16040202

**Published:** 2026-04-03

**Authors:** Parhesh Kumar, Ingharan Siddarthan, Catharine Kelsh Keim, Daniel K. Cho, John E. Rubin, Robert S. White, Rohan Jotwani

**Affiliations:** 1 Weill Cornell Medical College, Cornell University, New York, NY 10065, USA; pak4011@med.cornell.edu; 2 Department of Anesthesiology, Weill Cornell Medicine, Cornell University, New York, NY 10065, USA; ijs9006@nyp.org (I.S.); ckk9001@nyp.org (C.K.K.); dkc7002@nyp.org (D.K.C.); jer9173@med.cornell.edu (J.E.R.); roj9068@med.cornell.edu (R.J.)

**Keywords:** virtual reality, extended reality, digital twin, education, medical, image segmentation, artificial intelligence, image processing, computer-assisted, anesthesiology, anesthesia, epidural, personalized medicine

## Abstract

**Background/Objectives**: Simulation digital twins (DT) models that integrate patient-specific imaging with artificial intelligence (AI)-based segmentation and extended reality (XR) technologies are rapidly increasing in relevance in personalized medicine. While their clinical applications are expanding, their role as reusable educational tools and the technical pipeline utilized for their development remain incompletely characterized. This narrative review examines current approaches to digital twin creation and XR integration, illustrated by a scoliosis-specific proof-of-concept educational case study. **Methods:** A narrative technical review was conducted by identifying relevant search keywords within the fields of AI-based image segmentation, extended reality in medicine, and medical education based on the authors’ expertise and familiarity with the subject. PubMed, Google Scholar, and Scopus were searched for English-language studies published primarily between 2015 and 2025 addressing patient-specific three-dimensional modeling, AI-driven segmentation, and XR applications in spine, orthopedic, anesthesiology, and interventional care. A de-identified case of scoliosis is used to present a proof-of-concept example of this process of creating a simulated digital twin for the purpose of medical education in a recorded XR format. **Results:** Prior studies demonstrated benefits of patient-specific 3D models for anatomical understanding and procedural planning, while highlighting limitations in segmentation accuracy and workflow integration. Nevertheless, while DTs have traditionally served clinical roles in surgical planning or pre-procedural rehearsal, their pedagogical potential remains under-explored. In the proof-of-concept case study, AI-assisted segmentation enabled rapid creation of an anatomically detailed scoliosis digital twin that was incorporated into XR and used to produce a reusable, spatially anchored instructional experience focused on neuraxial access. **Conclusions:** AI-enabled digital twin models integrated with XR represent a promising approach for personalized, anatomy-driven medical education. Further evaluation is needed to assess educational outcomes, scalability, and integration into clinical training workflows.

## 1. Introduction

The convergence of advanced medical imaging modalities, artificial intelligence (AI)-driven segmentation, and immersive medical extended reality (XR; an umbrella term inclusive of virtual, augmented and mixed reality) technologies is opening a new frontier in personalized interventional care. At the core of this transformation lies the concept of the “digital twin (DT)”: a virtual replica of a patient’s unique anatomy, and, in advanced implementations, physiology that supports individualized treatment planning, simulation, and education [1,2,3,4].

There are two broad categories of DTs: one aimed towards “monitoring” and another targeted towards “simulation” [2,5]. Monitoring DTs require dynamic two-way communication between patient and twin, offering real-time data that can be used to alter treatment and monitor results [2]. On the other hand, simulation DTs are a one-way construct from patient to twin used primarily for modeling and planning therapies and interventions, serving as static snapshots of the patient with greater fidelity [2]. Monitoring DTs therefore extend beyond 3D reconstructions; they may integrate additional data streams and predictive modeling to simulate how interventions might affect an individual over time [6]. Three-dimensional (3D) patient-specific models generated from patient imaging in clinically procedural and educational contexts is the focus of this review, and would therefore be an example of simulation DTs.

In surgical and interventional contexts, it has been shown that interacting with simulation DTs can improve precision, anticipate anatomical challenges, and enhance decision-making, potentially reducing complications and optimizing outcomes [7,8,9]. When paired with XR, including virtual reality (VR) and augmented reality (AR), these models become interactive platforms. Clinicians and trainees can manipulate patient-specific anatomy in immersive, spatially accurate environments, rehearsing complex procedures prior to engaging with the patient [10]. The evidence for this practice continues to evolve in various procedural contexts. A systematic review of 19 studies encompassing 2157 pedicle screws demonstrated that augmented reality surgical navigation achieved superior screw placement accuracy compared to freehand, fluoroscopic, and intraoperative image-guided techniques, with statistically significant improvements across all comparators, and additionally results in significantly less intraoperative blood loss [8]. On a smaller scale, a case series from our group demonstrated that across six patients with anatomically challenging conditions, VR-assisted pre-procedural planning using patient-specific 3D models facilitated improved visualization of complex anatomy, enabled precise lead or needle placement in patients with prior surgical changes or malignancy, and in at least one case potentially prevented patient harm by identifying an alternative procedural approach [4].

Implementation of this vision demands careful attention to the technical pipeline: (1) image acquisition and conversion of radiologic image files (specifically Digital Imaging and Communications in Medicine [DICOM]) into 3D meshes; (2) application of AI-based segmentation and colorization pre-sets to generate a useable model; (3) integration of models into XR systems; (4) lesson conceptualization and execution with the model for medical education. Each step carries limitations and requires careful analysis for accuracy prior to affecting patient care [11,12]. Available literature captures aspects of each of these critical steps, but there remains a lack of clinician- and educator-accessible descriptions of the end-to-end technical pipeline required to transform patient imaging into reusable XR-based educational tools.

**Figure 1:**
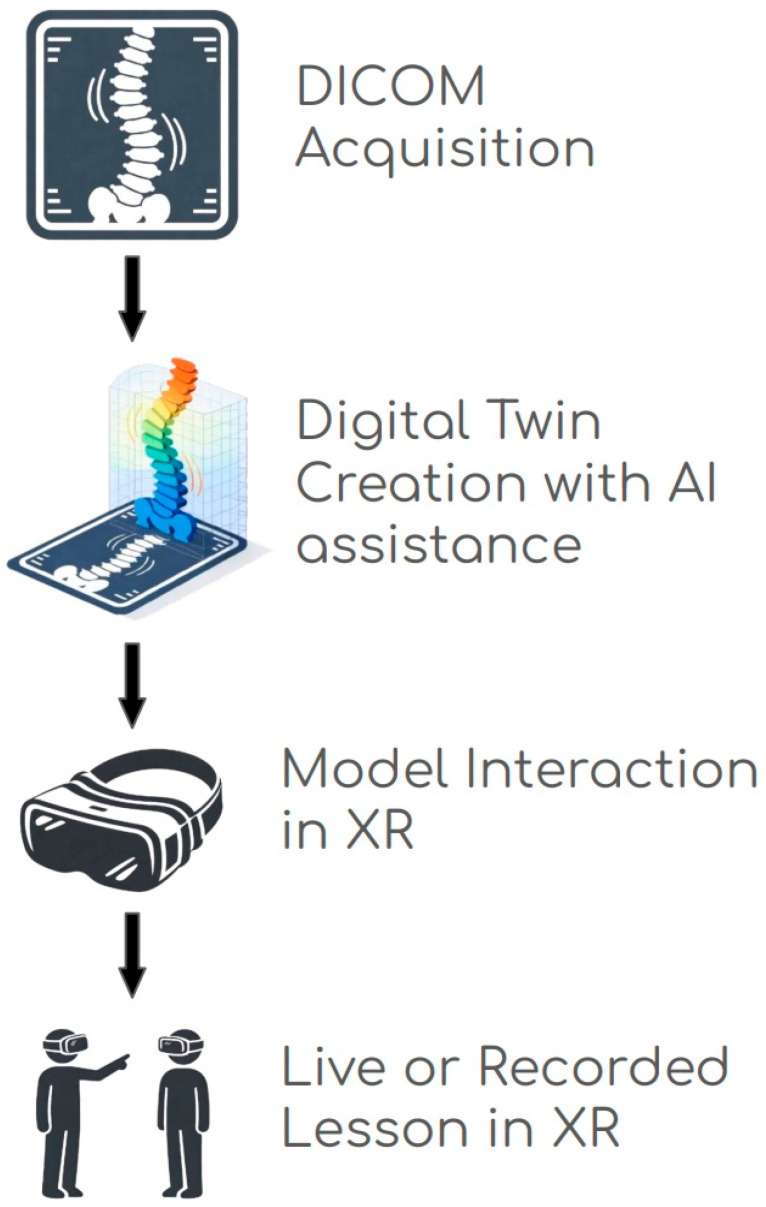
Key steps of the pipeline from individual patient imaging to patient-specific medical education. DICOM files of the images of patient/pathology of interest are identified. Using AI and manual optimization, the images are segmented and a DT is created. The model is viewed in XR and can be manipulated in a 3D space and interacted with using functions such as draw, rotation, splice, and zoom. A lesson is developed by an expert and delivered live or via recording to a learner.

This narrative technical review has three explicit aims: (1) to synthesize the current literature on AI-based segmentation and XR as they pertain to patient-specific 3D model creation (the role of imaging physics, density/tonal mapping, segmentation processes, colorization, and known constraints); (2) to describe the end-to-end technical pipeline through which patient imaging is transformed into reusable XR-based educational assets; and (3) to illustrate this pipeline through a bounded, proof-of-concept case example. These aims are complementary and intended to function together: the review provides the conceptual and technical framework, while the case example provides a concrete, worked demonstration of the pipeline in practice (Figure 1). By bridging imaging, AI segmentation, and XR visualization, this work aims to provide a clinician-accessible technical reference for those seeking to implement similar pipelines in educational and clinical contexts [13].

For the purposes of this manuscript, terms like “simulated digital twin,” “patient-specific 3D model,” and “XR model” are used interchangeably in various contexts based on the definitions above. We acknowledge that ‘digital twin’ is used variably in the broader literature, and that most of what is described here corresponds most precisely to a simulation digital twin (i.e., a static, patient-derived anatomical model) rather than a dynamic monitoring digital twin. Where the distinction is clinically meaningful, it is noted explicitly.

## 2. Materials and Methods

### 2.1. Narrative Review

We performed a narrative technical review of the literature to synthesize current approaches to patient-specific 3D modeling, AI-based segmentation, and extended reality (XR) and spatial media applications in clinical education. Literature was identified through targeted searches of PubMed, Google Scholar, and Scopus. Search terms included various combinations such as “digital twin” AND “3D model” OR “patient-specific model” AND “planning”, “AI segmentation” OR “machine learning segmentation” AND “image segmentation”, “segmentation” AND “computed tomography” OR “magnetic resonance”, “extended reality” OR “virtual reality” OR “augmented reality” OR “mixed reality”, “spine” OR “scoliosis” OR “vertebra” AND “interventional pain” OR “spine surgery”, and “virtual reality” OR “3D print” OR “anatomical model” OR “virtual model” AND “medical eduation” OR “training” OR “learning” AND “neurosurgical” OR “orthopedic” OR “anesthesiology”. This survey was supplemented by a manual review of reference lists from seminal works on core imaging physics and key technical articles.

Studies were selected based on their technical relevance, conceptual clarity, and instructional value for clinicians and educators. This approach aligns with narrative synthesis methodology for emerging and heterogeneous technological domains, prioritizing representative and foundational studies that illustrate the end-to-end development pipeline [12,14]. By focusing on both novel implementations and established technical primers, the review provides a conceptual framework for implementation of these emerging technologies [15,16].

The findings are structured into three primary categories: image modality fundamentals, technical aspects of 3D modeling and segmentation, and the integration of deep learning AI. An emphasis is placed on pitfalls that must be appreciated at each step to make an accurate and useful model. The review is intentionally focused on computed tomography (CT) images as the available literature studies this more consistently. As with all narrative reviews, study selection reflects the authors’ judgment and is not fully reproducible; this is a recognized limitation of the methodology and should be considered when interpreting the scope and representativeness of the findings.

### 2.2. Case Study: Development of Scoliosis-Specific Digital Twin Model

To illustrate the practical application of the reviewed technologies, we developed a proof-of-concept patient-specific digital twin model for a case of scoliosis managed at our institution. This proof-of-concept example is bounded in scope and is intended solely as a technical demonstration of the model creation pipeline; it does not constitute a validation study and makes no formal claims regarding educational outcomes. This case emphasizes the use of personalized healthcare models for use in training for interventional pain procedures. The patient was chosen based on the physicians discretion where it was believed 3D modeling would be impactful and beneficial in explaining scoliosis. Informed consent was obtained. All information about the patient’s record was deidentified, including DICOM images, and uploaded on HIPAA compliant cloud servers for any visualization.

The primary focus of this case study is the model creation pipeline, which integrates imaging acquisition, AI-assisted segmentation, and XR visualization to produce an accurate, interactive replica of the patient’s spine (Figure 1). High-resolution imaging data were acquired using CT scans in DICOM format. Model creation was performed using MedicalHolodeck software (MEDICALHOLODECK Inc., Zurich, Switzerland, latest version 2025), using similar methodology described in prior work [5,6]. Following DICOM import, HU calibration was performed to isolate bone tissue (targeting 150–800 HU), using real-time inspection of HU values to inform thresholding parameters.

Following HU adjustment, AI-driven segmentation was run via one-click activation, utilizing the TotalSegmentator (open-source totalsegementor, University of Basel, Switzerland) model pre-trained on annotated spinal CT datasets (at 0.6 mm resolution). The AI segmentation is loaded alongside the original DICOM data in the segmentation control panel, organizing detected structures into groups (e.g., bones, organs). It was particularly useful for removing static noise, artifacts, or irrelevant organs without altering HU selection or colorization—for example, in our model optimized for bone (150–800 HU range), liver tissue overlapping this spectrum was partitioned and hidden via visibility toggles (eye icon for hide/show) to focus solely on scoliosis-relevant vertebral structures.

After selecting the refined HUs and relevant structures, colorization was implemented using the segmentation transfer function in the control panel. Colorization was applied via lookup tables (LUTs) mapping HU ranges to intuitive hues; a black-to-amber gradient was used to emphasize bony depth and anatomical contrast.

This model creation process allowed us to take a 2-dimensional grayscale CT image and convert it to a 3-dimensional structure with a specific focus on bone tissue, which then allowed for real-time manipulation of anatomical structures, simulation of interventional pain procedures (e.g., epidural injections or facet joint blocks), and trajectory planning to anticipate challenges like rotated vertebrae or narrowed foramina.

### 2.3. Lesson Creation in Recorded Extended Reality

As a conclusion to the illustrative case example, a recorded educational session was created using the model’s spatial media features, demonstrating its application in a simulated classroom setting for higher-level training (Appendix A).

RXR (Recorded Extended Reality, developed by MedicalHolodeck) was used as a proprietary spatial capture methodology in the anatomy sandbox to enable the immersive recording of user interactions within a virtual environment.

Within the immersive XR platform, an experienced instructor (an interventional pain faculty member) navigated the model while delivering a spatial lecture focused on the challenges of neuraxial access in severe scoliosis. Using the RXR toolset, their movements, 3D drawings/markings, hand gestures, ray casting and voice were captured in real-time as a spatial object independent of the 3D scoliosis model and 2D assets such as de-identified radiographs of scoliosis.

The RXR recording was extracted from the HMD to a workstation laptop for post-production editing. The final edited product was reuploaded to multiple HMDs as a preserved interactive, spatially anchored media file, enabling asynchronous replay by trainees wearing standard VR headsets.

## 3. Results

Studies were selected based on direct relevance to the three technical categories structuring this review, with priority given to foundational works and state-of-the-art implementations that best illustrate the end-to-end pipeline. This purposive, non-exhaustive approach is consistent with narrative synthesis methodology.

### 3.1. Imaging Modalities for Model Creation

The creation of patient-specific 3D models from medical imaging has evolved with emerging, and now more accessible, technologies such as VR headsets and AI segmentation for a host of purposes from medical education, preprocedural planning, and even intra-operative use [15,17,18]. The process of creating these 3D models starts with the acquisition of high-resolution imaging data, grounded in the principles of imaging physics. In constructing models, CT excels at capturing bony structures via X-ray attenuation, while magnetic resonance (MR) provides superior soft tissue contrast via magnetic field interactions [16]. In CT, X-rays pass through the body, and the degree of attenuation is quantified to form cross-sectional images. The physics here involve the interaction of photons with matter, governed by the Beer-Lambert law, which describes exponential attenuation based on tissue density and atomic number [19]. Early CT scanners in the 1970s offered coarse resolutions, but modern multi-detector systems (e.g., 64-slice or higher) enable sub-millimeter voxels, producing DICOM files that serve as the raw input for modeling [20]. DICOM files represent the standard format for storing and transmitting medical images, such as radiographs, CT, and MR images; this format encompasses both the image data itself and essential metadata, including patient demographics, acquisition parameters, and equipment settings, facilitating consistent viewing, sharing, and processing across healthcare systems [16].

Upon importing a DICOM image into a software for 3D analysis, the density and tonal mapping are primary steps in translating physical attenuation into quantifiable values for model building. For CT, this is achieved via the HU, a linear scale calibrated such that water is 0 HU and air is −1000 HU, introduced by Sir Godfrey Hounsfield to standardize radiodensity across scanners [21]. Tonal mapping assigns grayscale values to these densities, with higher HU (e.g., bone at +300 to +1000 HU) appearing brighter, forming the basis for 3D visualizations in fields like orthopedics [22]. Radiologists leverage these differences in composition to visually distinguish tissues, enabling precise diagnostics and model inputs. In 3D modeling, HU implications are profound; they guide thresholding for tissue isolation, assigns material properties (e.g., denser HU for rigid bone simulations), and informs color/tonal rendering for realistic visualizations [20].

HU values exhibit variation due to factors like tube voltage, current, reconstruction algorithms, and patient-specific elements (e.g., body habitus), which can shift readings by 20–30% across scans [22]. Consequently, fine-tuning (e.g., window/level adjustments) is necessary to ensure model accuracy and avoid misrepresentation of tissue densities or even entire structures. Given that HU ranges correspond to specific tissue structures, DT models can use this to segment those tissues, isolating anatomical regions to build focused models. Within the field of spine care, such models are utilized to identify spine abnormalities such as vertebral compression fractures [23], estimate load mechanics within the spine [24], and assist image-guided spine surgery [25].

### 3.2. Technical Aspects of Image Segmentation and 3D Modeling

Image segmentation at its core is the process of identifying and separating different structures (such as bone, soft tissue, and fluid) on a pixel-by-pixel basis to enhance differentiation between these structures. Traditionally, before AI dominance, segmentation relied on manual or semi-automated methods. Model generation involves several steps: structure identification, anatomic segmentation and differentiation, and three-dimensional reconstruction [26].

Boundary delineation is the most critical step of image segmentation [27]. In spine imaging, for example, the borders of what denotes “bone” versus “soft tissue” versus “neural structures” need to be clearly defined for each slice of a scan to build an accurate and useful 3D model. Manual or semi-automatic methods involve contouring structures slice-by-slice, applying basic algorithms like region growing (starting from seed points and expanding based on HU similarity), edge detection (identifying boundaries via gradient changes), threshold adjustment (analogous to window adjustment to distinguish structures without losing clarity) and manual editing to fill gaps and edit mistakes made by automation [19,28]. The breakthrough in making this process more efficient was a series of innovations that focused on border delineation, such as watershed processing [29,30,31], and clever uses of statistical and predictive machine learning modeling [32,33]. Specifics of manual segmentation are beyond the scope of this review but are mentioned as strategies to keep in mind when critically examining DTs created using AI-based segmentation.

A way to accentuate the different tissue after the segmentation step is to allocate certain colors to ranges of HU to enhance visualization, mapping densities to hues for intuitive interpretation. This technique employs lookup tables (LUTs) to assign colors based on HU thresholds—for instance, rendering low-density fat in yellow (−100 to −50 HU), muscle in red (10–40 HU), and bone in white-to-blue gradients—evolving from monochromatic models to multi-hued representations that mimic real tissues [22]. In the field, colorization has been pivotal in radiology for tumor delineation and in surgery for layered anatomical guides; for example, in 3D-printed phantoms, differentiated hues simulate tissue interfaces, improving accuracy in procedures like airway management or vascular interventions [34,35].

The narrative is incomplete without acknowledging constraints to manual and automated segmentation [26,27]. Imaging artifacts, like motion blur in MRI or metal-induced distortions in CT, can skew density maps by 20–30%, compromising model fidelity—a nuance addressed in iterative scanner advancements [36,37]. Resolution limits in low-dose protocols introduce partial volume effects, blurring boundaries and causing volume errors >10% in thin structures, a trade-off for patient safety [22]. On a broader scale, the expertise required to create these models with fidelity and consistency, and to understand the shortcomings of automated processing, restricted access to specialists within the field [38,39]. Segmentation was limited to a slice-by-slice analysis that was prone to over-emphasizing differences that were ultimately artifactual or clinically insignificant [38]. In other words, the trees were analyzed in detail, but the forest was missed.

### 3.3. AI-Based Segmentation Using Deep Learning: Increasing Accessibility to 3D Model Creation

The advent of deep learning methods for image segmentation has revolutionized the field of image segmentation and modeling [26,27]. Following the implementation of earlier automated tools of segmentation, classical machine learning algorithms (encompassing many of the aforementioned model optimization techniques) dominated the field until very recently.

In 2015, authors Ronneberger et al. published the first example of a convolutional neural network (CNN) utilized for CT and MR segmentation, known as U-Net [40]. Classical machine learning relies on humans to define its rules and works best for structured datasets. Deep learning methods, however, can perform layers of calculations on unstructured data such as images and pixels. The U-Net CNN determines its own understanding of borders, textures, and intensities across all slices of a scan, based on a smaller subset of human-verified analyses that serve as “ground truth”. This was quickly followed by V-Net in 2016, which introduced “volumetric segmentation”, the generation of 3D structures from 2D images [41].

AI-generated segmentations are verified against the ground truth using several measures of agreement, most often the Dice similarity coefficient. A coefficient closer to 1.0 represents an increasingly precise model compared with models segmented manually by expert humans [42,43]. U-Net remains one of the most widely used frameworks for current modeling software due to its ease of use, replicability, and fidelity.

AI significantly enhances model generation by automating complex tasks, such as artifact correction and integrating scans of different quality and age [44]. Deep learning models also do this quickly; literature suggests CNNs achieve high accuracy and efficiency gains of 70% [45,46]. However, challenges persist, including overfitting to biased datasets (errors > 15%), GPU-dependent computation, lack of transparency, and artifact sensitivity, necessitating hybrid approaches for ethical deployment [47,48,49,50,51,52].

The current era is one of optimization of these networks to reduce bias and increase generalizability without compromising accuracy [53]. In 2021, nnU-Net (“no-new” U-Net) revolutionized AI segmentation by expanding generalizability and increasing accessibility for clinicians [53,54]. This network automatically configured parameters for model creation based on the training dataset, and dramatically reduced the risk of model breakdown when applied to images outside the dataset. One such model created using nnU-Net is TotalSegmentator, an open-source model for volumetric segmentation that robustly segments major anatomic structures in a variety of body CTs [55,56]. This model serves as the engine for Medical Imaging XR (© MEDICALHOLODECK Inc., Zurich, Switzerland) utilized for the following illustrative case. The technology was recently applied to plan and perform advanced pain procedures on patients with complex prior surgical histories or altered anatomy due to malignancy [4].

Steps now involve data preparation (annotating images), model training/validation, and deployment, reducing time to minutes. Implications include enhanced accuracy (e.g., handling artifacts better), scalability for large datasets, and broader accessibility via open-source tools like MONAI, democratizing modeling for non-experts [16]. In education, segmentation serves as a practical use case for teaching anatomical precision; for instance, interactive tools allow learners to apply thresholds and refine models, building skills in image analysis and fostering an understanding of tissue differentiation, as demonstrated in immersive training programs [18]. For novices, this step demystifies data: Import a DICOM, apply thresholds, and iterate refinements, a process that has shifted from labor-intensive (hours per model) to streamlined (minutes with presets) [45].

Some more novel models are being developed that can expand beyond CT and MR images to segment photos of dermatologic lesions and images from bronchoscopy with great external validity [57]. Advanced volumetric segmentation has incredibly useful applications especially related to obtaining accurate measurements of structures without the limitation of 2D slices, including measurement of true Cobb angles in scoliosis [58] and measurement of ligamentum flavum thickness in preparation for percutaneous image-guided lumbar decompression [59]. The following case study explores an application of volumetric segmentation: the creation of an accurate model of severe scoliosis used to educate anesthesiologists on the placement of a landmark-guided epidural catheter.

### 3.4. Developing XR Educational Media from Digital Twin Models: An Illustrative Case of Scoliosis Modeling

To illustrate the practical application of the technologies reviewed, we developed a DT of a patient with severe scoliosis and used it to create an immersive educational module (Appendix A). This process demonstrates how volumetric segmentation and XR technologies can transform a static imaging dataset into a dynamic, reusable spatial teaching asset.

Application of the pipeline to a scoliosis case yielded an anatomically detailed, AI-segmented 3D model of the thoracolumbar spine, rendered within a VR environment (Meta Quest 3) using MedicalHolodeck’s Medical Imaging XR platform. The underlying segmentation model (TotalSegmentator) has published benchmark accuracy of mean Dice similarity coefficient 0.943 (95% CI: 0.938–0.947) across 104 anatomic structures including 59 bones [55], providing the accuracy reference for this pipeline in the absence of independent case-level validation. An expert instructor used the RXR spatial capture toolset to record a guided educational session within the VR environment, incorporating spinal landmark identification, neuraxial access technique, and 3D annotation of pathologic anatomy relevant to severe scoliosis (full lesson transcript in Appendix A).

The session was recorded as an RXR media file—capturing voice, hand motion, model manipulation, and spatial references in real-time. Post-processing was conducted using proprietary RXR editing tools, allowing for trimming, layering of visual assets (e.g., X-rays, trajectory diagrams), and spatial editing of the instructor’s avatar and annotations.

**Figure 2:**
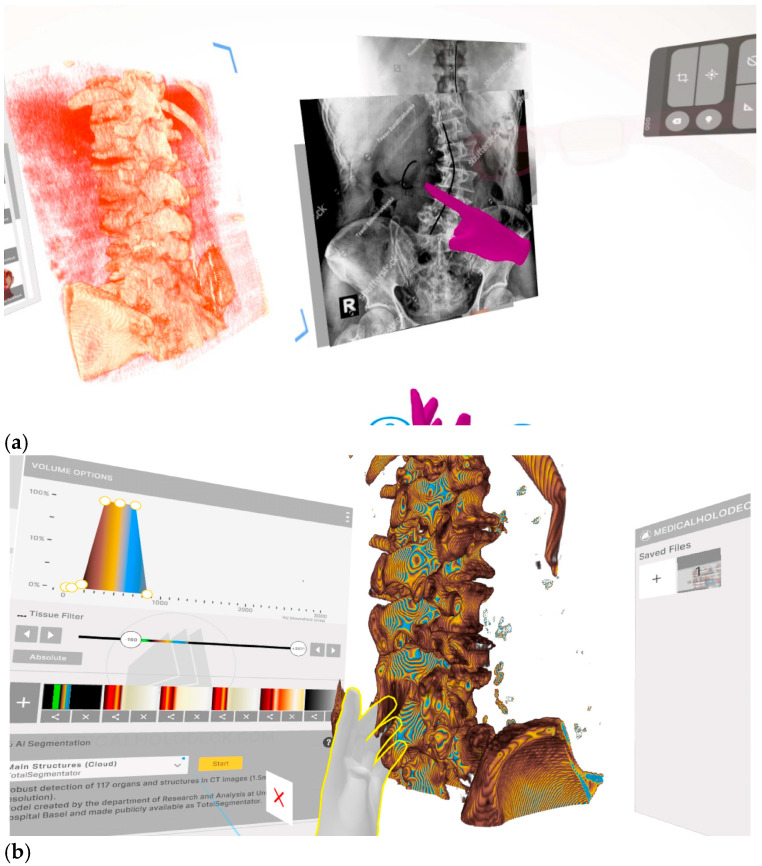

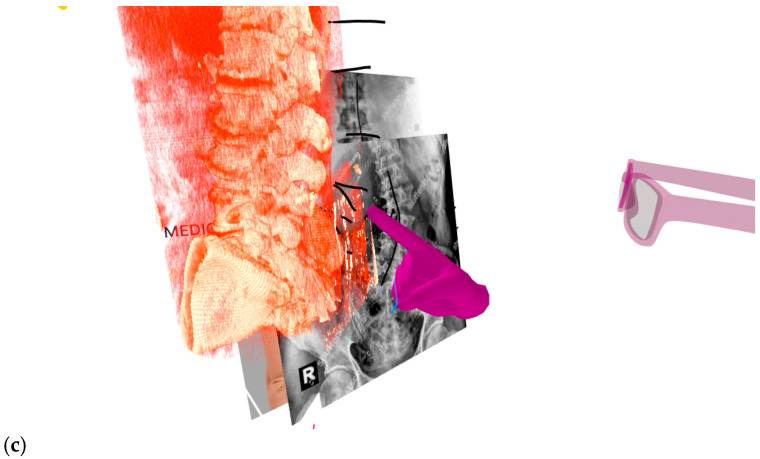
Images from XR Recorded Session. (**a**–**c**). Screenshots from a live session of the learner’s point of view interacting with the RXR lesson. The image was created using a virtual reality headset which shows the learner seeing imposed 3D holograms and 2D pictures in an otherwise blank background. (**a**) Shows a sample from the recorded lesson where the learner stands in the direct field of view of the instructor looking at the 3D rendered scoliosis model with side-by-side images of X-ray scoliosis anatomy. The magenta hands in the picture represent the recorded instructor drawing on the digital assets as the lesson plays. (**b**) Shows a still image of the learner holding and manipulating the 3D scoliosis model using their virtual reality remote controllers. In the background there is a 2D plane showing options for volumetric manipulation for color and HU ranges, along with options for automatic AI segmentation. (**c**) Shows the learner able to look at the RXR lesson from different angles beyond the point of view of the instructor at the time of filming. The magenta glasses and hands represent the embodiment of the instructor visualized for the learner. The instructor is making the obliquity of the spinous processes on the scoliosis model to provide insight into pathologic anatomy.

The final product was a self-contained, repeatable spatial learning experience viewable on any compatible VR headset (Figure 2). Trainees could enter the environment, observe the expert’s spatial references, and explore the digital twin model independently or guided by the recorded narration. Unlike traditional 2D video instruction, this XR module preserved the instructor’s spatial reasoning and tactile explanations tied to the digital assets, creating an interactive anatomy lesson anchored in a real patient’s case. In addition, learners could rewind the lesson, or manipulate the model themselves to reinforce anatomical comprehension.

## 4. Discussion

This study illustrates the evolving utility of patient-specific 3D models in medical education, particularly when combined with immersive technologies like XR. While DTs have traditionally served clinical roles in surgical planning or pre-procedural rehearsal, their pedagogical potential remains under explored [3,60]. Our case demonstrates how a volumetric scoliosis model (derived from patient data) can be transformed into a reusable, spatially-rich educational asset using XR, offering a compelling proof-of-concept for personalized, high-fidelity teaching.

Preoperatively, DTs offer clinicians unprecedented insight into patient-specific anatomy. As shown in multiple studies, volumetric modeling improves procedural accuracy, facilitates trajectory planning, and reduces complications in spine surgery and interventional pain management [12,15,18]. However, the fidelity of these models relies heavily on imaging inputs and the accuracy of segmentation. AI-based tools such as TotalSegmentator and nnU-Net have shown promise in automating complex segmentations with high Dice coefficients, making them increasingly viable for real-time clinical applications [45,46].

Intraoperatively, the application of simulated digital twins has begun to support augmented and mixed reality overlays during surgery, although most XR implementations remain limited by hardware constraints and workflow integration challenges [1,61]. Notably, few XR tools allow for dynamic, patient-specific updates during procedures, limiting intraoperative adaptability. Despite these challenges, early adopters report improvements in anatomical orientation, especially in minimally invasive or anatomically distorted cases, such as those involving scoliosis [52,62].

Pedagogically, XR tools show promise. Studies in orthopedic and anesthesiology education have demonstrated enhanced spatial understanding and procedural recall when learners train using immersive 3D models compared to traditional 2D modalities [7,51,52]. 3D printed models have been successfully used for anatomy education and procedural education for thoracic epidural placement by anesthesiology residents [59,63]. However, few implementations have successfully integrated patient-specific data into persistent, interactive training modules.

One of the most significant contributions of this approach is the transition from static anatomical representations to dynamic, spatially anchored instruction [60]. Traditional medical education relies heavily on didactic formats and 2D resources, which may not fully convey the complexity of patient-specific anatomy, especially in cases like severe scoliosis where rotational deformities are difficult to appreciate without 3D manipulation [50,64].

While many fields of medicine have explored the benefits of 3D instruction, the concept of virtual 3D models for education is still in its infancy [65,66]. Within the last few years, however, there has been excitement in many medical disciplines on the potential for education in XR, ranging from procedure anatomy education to training in surgical skill [67,68,69]. The XR method allows instructors to embed their expertise directly within the spatial environment, preserving both verbal explanations and hand-based anatomical cues for learners to access asynchronously. This approach is theoretically consistent with the cognitive benefits of spatial memory, though formal assessment of retention and anatomical reasoning in this context remains a subject for future study.

From a technological standpoint, using XR recordings as a pipeline leverages the increasing maturity of AI-based segmentation and digital twin interfaces. TotalSegmentator enabled rapid and accurate delineation of spinal structures in our case study, reducing manual input and variability. Importing this model into Holodeck’s XR platform and recording the instructional session via RXR created a multimodal artifact or “spatial memory” (part anatomy model, part expert lecture) that can be reused across cohorts and global settings. Such recordings may offer a scalable solution to specialist knowledge transfer, especially in settings where expert instruction is limited, or asynchronous learning is required [5,43,70].

Our preliminary deployment among interventional pain procedures reflects the underlying expertise of the authors of this manuscript and a specific proprietary software ecosystem. Structured outcomes (e.g., knowledge retention, procedural performance) were not formally assessed and remain a key next step for evaluating educational impact. Accordingly, the case example presented here should be interpreted as an illustrative proof-of-concept technical demonstration of the proposed pipeline rather than as a formal validation study of educational outcomes. Similarly, while our case used spine anatomy and neuraxial technique as an exemplar, the method is readily generalizable. Cardiac anatomy learning in the setting of congenital abnormalities, peripheral nerve navigation, and complex airway management may all benefit from recorded spatial instruction built upon patient-specific DTs.

There are important limitations. First, the fidelity and effectiveness of the experience depend on model quality; segmentation errors or poor imaging inputs can propagate into the educational content. The scoliosis case was selected as an illustrative workflow demonstration rather than a validation study, and formal Dice-based comparison against manually segmented ground truth was beyond the scope of this proof-of-concept. Second, while XR headsets are increasingly available, institutional access and user readiness may vary, and equity of access must be considered as this approach scales. Third, for this particular illustrative case study, the use of spatial assets depends on proprietary RXR technology, and while RXR files can be transferred between headsets, interoperability is currently limited to devices running the underlying MedicalHolodeck software ecosystem. This is a recognized challenge across the XR field more broadly; open standards such as OpenXR and WebXR offer a path to overcome this fragmentation, enabling interoperability across devices and supporting scalable deployment in teaching hospitals [71]. The motion-capture methodology underlying RXR is conceptually analogous to spatial recording features being incorporated across a growing range of XR platforms in gaming, simulation, and education, and future iterations of these tools are likely to support greater cross-platform portability of spatial educational content. Fourth, the use of AI or “one-click” segmentation approach via TotalSegmentor used in this case study invites known constraints of models trained predominantly on normative datasets (discussed in Section 3.2). Normative training datasets may not adequately capture the full spectrum of severe spinal deformity. In cases of significant scoliosis, rotational deformity, or prior instrumentation, AI segmentation models may face compounding challenges: vertebral boundaries deviate substantially from expected anatomical positions, and metallic implants introduce CT artifacts that can severely limit image quality and tissue differentiation, even with advanced artifact reduction techniques. In the presented case, the absence of prior instrumentation meant these limitations were less clinically impactful; however, users applying this pipeline to post-surgical patients may result in errors and need for manual correction. Nevertheless, the development of pathology-specific training datasets represents an important direction for future work in this space. Lastly, spatial memory as a pedagogical format remains novel and warrants further usability studies to determine best practices for instructional design within spatial media both at the instructor and student level through metrics like formal knowledge retention assessment, procedural performance metrics, usability testing, and scalability evaluation across institutions and specialties. Future studies will need to critically examine the effectiveness of this educational modality and its reception by learners [26,62,71].

In summary, patient-specific 3D models integrated with AI-based segmentation and XR technologies represent a technically accessible and promising approach for anatomy-driven medical education. The pipeline described here offers a practical framework for clinician-educators seeking to translate patient imaging into reusable instructional assets. Formal evaluation of educational outcomes, scalability, and learner engagement remains an important next step before broader adoption can be recommended [1,3]. By embedding expert spatial reasoning into persistent 3D learning environments, this technique was shown as a proof-of-concept for use in personalized medicine and supports the development of next-generation curricula across medical specialties.

## Data Availability

The original contributions presented in this study are included in the article. Further inquiries can be directed to the corresponding author.

## Appendix A. Scoliosis Lesson Transcript

**I. 0:00–0:30 —Opening & Objectives** “Welcome, everyone. In the next few minutes, we’re going to use extended reality to understand how scoliosis changes spinal anatomy, and how those changes affect our approach to neuraxial placement. Scoliosis is common and presents multiple challenges such as distorted surface anatomy and deviation of the epidural space. These differences can mean that traditional approaches to neuraxial placement simply don’t work. By the end of this session, you should be able to recognize physical exam findings that provide clues to the underlying anatomic distortion, explore that distortion on 2D X-ray and in 3D, and integrate this information to plan a safer, quicker, and more effective needle trajectory.”
**II.0:30–2:00—Predicting Underlying Anatomy by Physical Exam** “The first step to successful neuraxial placement in the scoliotic patient is identifying the direction of the curve. Let’s begin with an external view of our patient. Sometimes this may be the only information we have. This is especially true for our obstetric patients, many of whom will not have had imaging prior to neuraxial placement. Perhaps you can tell just by looking at our patient that her curve has a convexity pointing to the right. But if you can’t tell just by looking at the spine, there are other physical findings you can look for that can provide clues to the direction of the curve. For instance, in our patient, you may be able to appreciate that her right shoulder is slightly higher than the left, and that her scapula is much more prominent on the right than the left. You may also notice that this area, called the “waist triangle,” is more straight on the convex side. This “triangle” is more acute on the concave side, where you can see these skin folds, which are absent on the convex side here. So, your first step in planning your needle entry and trajectory is determining the direction of the curve. In an ideal world, the spinous processes are visible, or can be palpated, to “map out” this curve. But, if you’re truly lost, look for these signs to give you a clue: higher shoulder on the convex side, more prominent scapula on the convex side, flattened waist triangle on the convex side.”
**III. 2:00–3:00—Using X-Ray to Understand Vertebral Rotation** “Now, scoliosis isn’t just a lateral curvature of the spine, but it also involves rotation of the vertebral bodies. In other words, it’s not a two-dimensional problem; it’s a three-dimensional problem. Nevertheless, let’s first bring up our 2D AP X-Ray to try and visualize this. Anatomically, the spinous processes point towards the midline (concave-side), and the vertebral bodies rotate towards the convex-side of the curve. In other words, the midline of the epidural space deviates toward the convex aspect of the scoliotic curve relative to spinous processes. You can appreciate this on X-Ray. Notice how the spinous processes point more towards the midline, and the vertebral bodies are angled away from midline towards the convex side. To convince yourself, notice how the pedicle on the convex side appears more medial on the X-ray—that’s your radiographic clue to where the spine is rotating. Now because of this rotation, there is an asymmetric narrowing of the interspaces. Again, looking at our 2D AP X-ray, perhaps you can see that the interlaminar spaces here are larger on the convex aspect of the curve. But as we said, scoliosis is a 3D problem, so let’s bring up our 3D model to visualize this better…”
**IV. 3:00–4:30—XR Exploration of 3D Anatomy** “This is our 3D model, which was created using CT images of an actual scoliotic spine. You can rotate the spine to appreciate the torsion of the vertebrae. Notice how the pedicles on the convex side appear more ‘open,’ while those on the concave side narrow and rotate away from you. It may be more obvious to you here than on X-ray that the interlaminar spaces on the right (convex side) are larger and more open than on the left. You can also notice here that the angle between the spinous and transverse process on the right side or convex side is much wider. Overall, you can appreciate there is just more room on the convex side. Now let’s revisit the vertebral rotation we discussed earlier. As we fade the posterior elements, watch how the true spinal canal drifts laterally relative to the skin. We can even better visualize that vertebral rotation if we rotate our model to an axial orientation. Looking at these axial cross-sections starting from the sacrum moving superiorly, you can see that as the degree of the curve increases moving upward, so too does the lateral rotation of the vertebral bodies. Now that we have a grasp on lateral rotation, vertebral rotation, and the interlaminar spaces, we can plan our approach to the epidural space…”
**V. 4:30–6:30—Applying the Anatomy to Epidural Technique** “Using an axial cross-section of our 3D model, lets first look at why traditional approaches to the epidural space might not work. If we start at the apparent midline of the spine, which you can imagine is somewhere medial to the spinous process, and aim for a trajectory that is perpendicular to the back, you can see our projected needle path will end up nowhere near the epidural space. Alternatively, if we start at the new midline at the spinous process and aim for a trajectory that is perpendicular to the back, again we end up meeting bone early and missing the epidural space. Now take a moment to think about the most direct path to the epidural space and how you would plan your needle trajectory… (pause) I want to cover two techniques, show you the projected needle path of each, and briefly discuss why one technique might be more successful than the other. The first is the midline approach. The needle enters the skin just above the spinous process and is advanced at an angle towards the convex side. You should feel engaged in ligament much like a regular epidural. The second technique is a modified paramedian approach. The needle enters the skin lateral to the spinous process on the convex side and is advanced perpendicular to the back. This may offer a straight shot directly into the epidural space, or if lamina is contacted, the needle can be walked cephalad until you reach the interlaminar space. Use incremental advancement, and expect asymmetry in resistance As you look at this, ask yourself: ‘Given this patient’s anatomy, what approach would give me the greatest margin for success?’ With the midline approach, to a certain extent you are guessing how much to angle your needle, but because you are midline you should have good tactile feedback and feel engaged if you are on the right track. Alternatively, the paramedian approach offers the advantage of approaching the epidural space where the interlaminar space is most open, and in severely scoliotic spines may be the only feasible path to the epidural space.”
**VI. 6:30–7:00—Summary & Takeaway Pearls** “To close, here are the three major takeaways: First: In order to plan your needle path, visualize the curvature of the spine beneath the skin. In the absence of imaging, spend time palpating or looking for other physical clues. Second: Remember, relative to the spinous process, the epidural space is shifted laterally towards the convex side of the curve due to vertebral rotation. Third: Visualize your intended needle trajectory using our 3D image as a mental model. Combining 2D radiographic interpretation with 3D spatial understanding—like we’re doing in XR—dramatically improves your ability to anticipate challenges and adjust your technique. Before we finish, consider one final question: ‘Based on today’s lesson, how will you plan your neuraxial technique the next time you encounter a patient with scoliosis? (Pause for answers) “Great work—this concludes the XR module. Feel free to revisit any of the 3D models or radiographs to reinforce your mental map.”
